# Asymmetric distribution of Spalt in *Drosophila* wing squamous and columnar epithelia ensures correct cell morphogenesis

**DOI:** 10.1038/srep30236

**Published:** 2016-07-25

**Authors:** Wenqian Tang, Dan Wang, Jie Shen

**Affiliations:** 1 Department of Entomology, China Agricultural University, 100193 Beijing, China

## Abstract

The *Drosophila* wing imaginal disc is a sac-like structure that is composed of two opposing cell layers: peripodial epithelium (PE, also known as squamous epithelia) and disc proper (DP, also known as pseudostratified columnar epithelia). The molecular mechanism of cell morphogenesis has been well studied in the DP but not in the PE. Although proper Dpp signalling activity is required for proper PE formation, the detailed regulation mechanism is poorly understood. Here, we found that the Dpp target gene *sal* is only expressed in DP cells, not in PE cells, although pMad is present in the PE. Increasing Dpp signalling activity cannot activate Sal in PE cells. The absence of Sal in the PE is essential for PE formation. The ectopic expression of *sal* in PE cells is sufficient to increase the PE cell height. Down-regulation of *sal* in the DP reduced DP cell height. We further demonstrated that the known PE cell height regulator Lines, which can convert PE into a DP cell fate, is mediated by *sal* mis-activation in PE because *sal-RNAi* and *lines* co-expression largely restores PE cell morphology. By revealing the microtubule distribution, we demonstrated that Lines- and Sal-heightened PE cells are morphologically similar to the intermediate cell with cuboidal morphology.

Cell shape changes occur throughout various developmental processes, such as *Drosophila* gastrulation^1^,^2^ and neural tube formation in vertebrates^3^,^4^. Reorganization of the microtubule cytoskeleton contributes to cell shape changes^5^,^6^,^7^ and thereby regulates the morphogenesis of tissues or organisms. In the *Drosophila* wing imaginal disc, all cells have a cuboidal shape in both cell layers in the 1^st^ and early 2^nd^ instar stages. During the late 2^nd^ and 3^rd^ instar stages, one layer of the cells is flattened to squamous epithelium (also known as peripodial epithelium, PE), whereas the opposite layer of cells elongates to columnar epithelium (also known as disc proper, DP). The intermediate cells at the wing disc margin linking the PE and DP exhibit a cuboidal shape^8^,^9^. Thus, the wing disc is a sac-like structure composed of two opposing cell layers^10^,^11^.

Several signalling pathways have been implicated in epithelium morphogenesis in the wing disc. Decapentaplegic (DPP) signalling activity is required to ensure the correct architecture of DP epithelium^12^,^13^. Dpp activity via Rho1 promotes the elongation of DP cells in a cell-autonomous manner^9^. A T-box transcription factor Optomotor-blind (Omb), one of the Dpp targets, is expressed in a gradient manner along the anterior-posterior axis to ensure correct DP morphology^14^. Wingless signalling regulates the apical cell circumference of DP cells by a proximo-distal gradient^15^. Together with Vestigial (Vg), Wingless signalling maintains DP cell elongation during the late larval stage^16^. DPP signalling is involved in PE cell morphogenesis. Mutant clones lacking Dpp receptor activity cannot form in the eye PE and display reduced size and aberrant shape in the wing PE^17^. When Daughters against Dpp (Dad), a suppressor of DPP signal transduction, is overexpressed in the wing PE, a portion of the wing PE cells are elongated^8^.

In addition to Dpp signalling, Hedgehog (Hh) signalling and other putative transcription factors are also important for PE morphogenesis. Clones lacking Hedgehog (Hh) function in the eye PE are smaller than their wild-type twinspots, and the anterior ventral domains of both PE and DP are lacking in these clones^11^. Similarly, the wing disc is smaller in size and fails to form the PE in *hh* mutants^8^. In addition, the putative transcription factor Lines (Lin) promotes elongation of the PE and converts these cells into a DP cell fate. Lin is also necessary for the expression of the Dpp target gene *spalt* (*sal*)^18^.

Although appropriate Dpp signalling activity is necessary for PE morphogenesis^8^,^17^, the downstream mediators of Dpp signalling remain unknown. Here, we report that the known Dpp target genes, including *sal* and *omb*, are not expressed in PE. The absence of *sal* but not *omb* insures correct PE morphogenesis. The ectopic expression of *sal* in the PE is sufficient to elongate PE. Lin requires *sal* function to elongate the PE because suppressing *sal* can rescue the phenotype induced by *lin* ectopic expression. When the microtubule distribution is visualized, Sal-elongated PE cells are morphologically similar to cubically intermediate cells that normally link the PE and DP layers.

## Results and Discussion

### *sal* is not expressed in the PE

**Figure 1:**
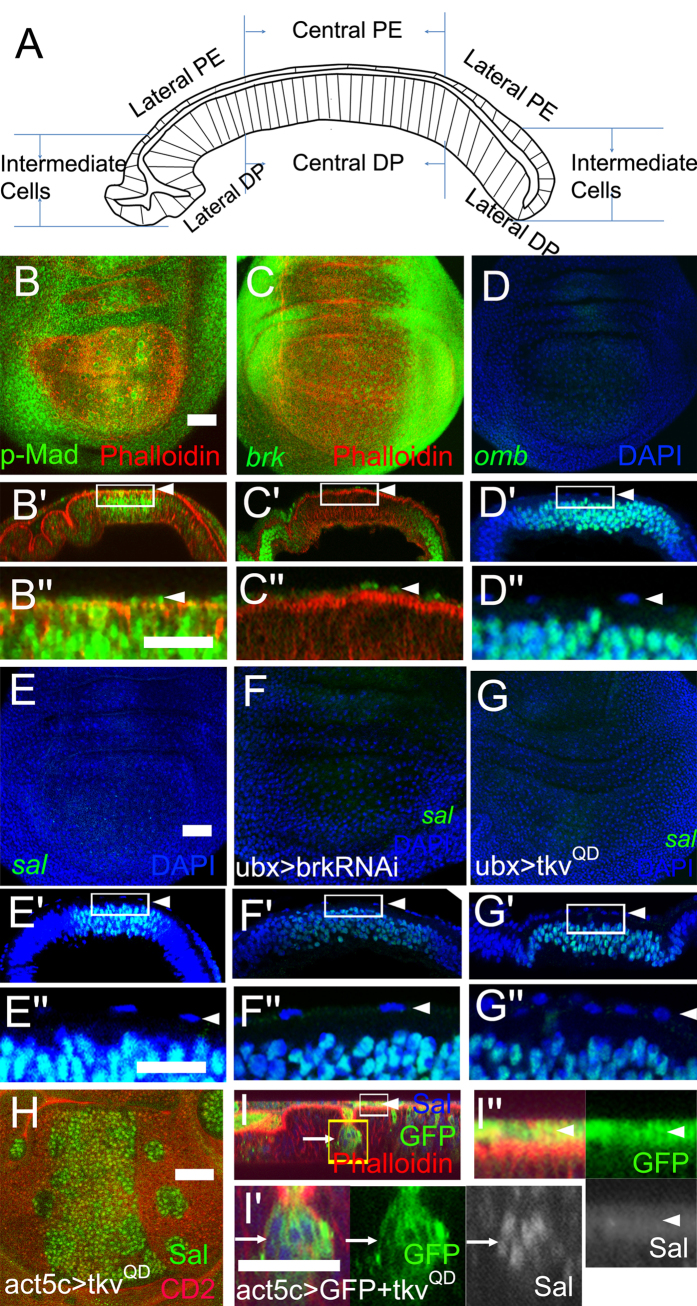
*sal* is not induced by Dpp signalling in the PE. In this and subsequent figures, late L3 wing discs are used, otherwise developmental stage is indicated. *x-y* views are oriented with dorsal up and anterior left. Cryosections (*x-z* views) along the dorsal-ventral boundary are oriented with apical up and anterior left. Scale bars are 50 μM. In *x-y* views of (**B–G**) panels are focused on the PE plane. H is focused on the DP plane. Others are *x-z* views of the wing disc. (**A**) Schematic drawing of wing disc structure in *x-z* view. The wing disc is a sac-like structure composed of two opposing cell layers with apical sides facing an internal lumen. (**B**–B″) pMad staining in the wing disc. Note that pMad is detected in the PE (arrowheads) and the DP. Boxed region in B′ is presented at a higher magnification in B″. (**C**–C″) *brk* expression pattern in the wing disc. Note that *brk* is transcribed in both the PE (arrowheads) and the DP as reveal by the *brk*-*lacZ* reporter. Boxed region in C′ is presented at a higher magnification in C″. (**D**–D″) *omb* expression pattern in the wing disc. Note that *omb* is only transcribed in the DP, not the PE (arrowheads), as revealed by the *omb*-*lacZ* reporter. Boxed region in D′ is presented at a higher magnification in D″. (E-E″) *sal* expression pattern in the wing disc. Note that *sal* is only transcribed in the DP, not in the PE (arrowheads), as revealed by the *sal*-*lacZ* reporter. Boxed region in E′ is presented at a higher magnification in E″. (**F**–F″) Expression of *brk-RNAi* does not induce Sal in the PE (arrowheads). Boxed region in F′ is presented at a higher magnification in F″. (**G**) Expression of *tkv*^*QD*^ does not induce Sal in the PE (arrowheads). Boxed region in G′ is presented at a higher magnification in G″. (H-I″) Expression of *tkv*^*QD*^ in clones induced Sal in the DP (arrow) but not in the PE (arrowhead). Boxed regions in I are presented at higher magnifications in I′ (a DP clone, arrow) and I″ (a PE clone, arrowhead).

The wing disc is a sac-like structure composed of PE, DP, and intermediate cells linking the PE and DP. An *x-z* view of the wing disc is schematically presented in Fig. 1A. To investigate the potential role of Dpp signalling in PE morphogenesis, we first revealed the distribution of Dpp signalling activity in late 3^rd^ instar (L3) wing imaginal discs in both the *x-y* and *x-z* views. Using an antibody of phospho-Mothers against dpp (pMad)^19^,^20^ to reveal Dpp signal transduction activity, we found that Dpp signal transduction was ubiquitously present in both the PE and DP (Fig. 1B–B″)^21^. The pMad level was relatively reduced in the central PE compared with the central DP (Fig. 1B′,B″, arrowheads). Then, we detected Dpp target gene expression patterns in the PE. The main Dpp target genes are *brinker* (*brk*), *omb*, and *sal* in the L3 wing discs^22^,^23^,^24^. *brk* was transcribed in both the PE and DP, with a relatively weaker level in the PE, as indicated by a *brk*-*lacZ* reporter (Fig. 1C–C″, arrowheads). However, both *omb* and *sal* were transcribed only in the DP, not in the PE (Fig. 1D–E″, arrowheads). These data indicate that the Dpp target genes *omb* and *sal* are asymmetrically expressed in the PE and DP. Brk is also a repressor of other Dpp target genes, including *sal* and *omb*, and thereby restricts their expression domains to the medial DP region^24^,^25^. The presence of Brk in the PE might be a direct cause of the absence of Omb and Sal in the PE. To assess this possibility, *brk-RNAi* was expressed in the PE. Sal expression was not detectable in the central PE (Fig. 1F′,F″, arrowheads). The efficiency of *brk-RNAi* was demonstrated by the elevation of Brk targets Omb and Sal in lateral wing discs of *C765*>*brk-RNAi* (data not shown). To further confirm that Dpp signalling cannot induce *sal* expression in the PE, a constitutive active form of the Dpp receptor *tkv*^*QD*^was expressed in the PE. Sal was not induced in central PE (Fig. 1G–G″). When *tkv*^*QD*^clones were generated, Sal was induced only in clones within the DP (Fig. 1H,I,I′) and not in clones located in the PE (Fig. 1I,I″). Similarly, Omb was not induced in clones located in the PE (Fig. S-1B, arrowhead). Ubiquitous expression of *tkv*^*QD*^failed to induce Omb in the PE (Fig. S-1C′,C″, arrowheads). These data demonstrate that Dpp signalling cannot activate *omb* and *sal* in PE.

**Figure 2:**
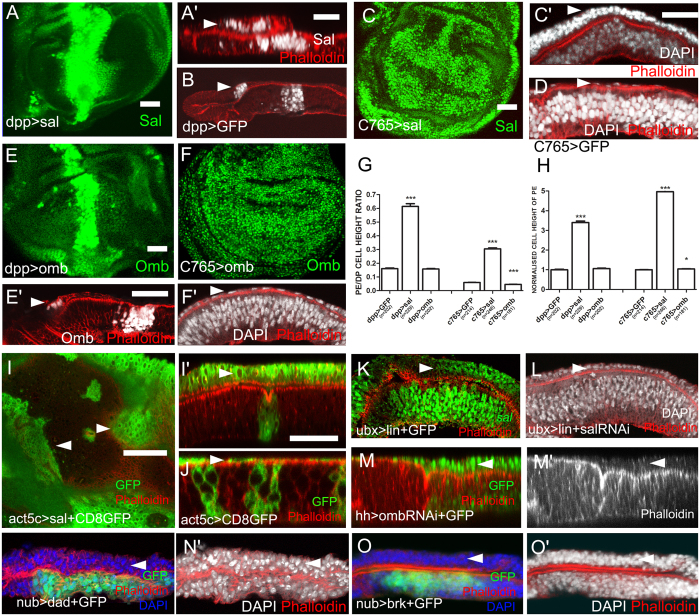
Ectopic *sal* expression elongates PE. (**A,C,E,F,I**) are *x*-y views, and the remaining images are *x*-z views. (**A**) Ectopic expression of *sal* in the *dpp-Gal4* domain induces elongation of lateral PE height (A′, arrowhead) compared with the control (**B**, arrowhead). (**C**) Ubiquitous expression of *sal* elongates PE height (C′, arrowhead) compared with the control (**D**, arrowhead). Note that the density of DAPI-stained cell nuclei in the PE is considerably increased compared with control. We compared central (flat) cells in *C765-Gal4* experiments (**C**) and lateral (less flat) cells in *dpp-Gal4* experiments (**A**). (**E**) Expressing *omb* in the *dpp-Gal4* domain does not elongate lateral PE height (E′, arrowhead). (**F**) Ubiquitous expression of *omb* does not elongate PE height (F′, arrowhead). (**G**) The statistics of Image-J program calculated ratio between PE and DP cell heights within one wing disc. (**H**) The statistical diagram of normalized PE height ratio between gene manipulations and corresponding control. In both (**G**,**H**) means ± SEM indicated by *** are significantly different (pairwise comparison of t-tests, p<0.0001). (**I**) Clones co-overexpressing *sal* and CD8-GFP cell membrane marker. Clones in the PE exhibit overgrowth (arrowheads) and elongation of PE height (I′, arrowhead). (**J**) Control clones expressing CD8-GFP. Arrowhead indicates a PE clone. (**K**) Expression of *lin* in the PE leads to the activation of *sal* transcription in the PE (arrowhead) as revealed by the *sal-lacZ* reporter. (**L**) Co-expression of *salm-RNAi* with *lin* largely rescues PE height. (**M**) Suppression of *omb* expression in the posterior DP has a non-autonomous effect on PE height. Arrow indicates PE elongation. (**N**) Expression of *dad* in DP induces non-autonomous PE elongation (arrowhead). (**O**) Expression of *brk* in DP induces non-autonomous PE elongation (arrowhead).

The expression patterns of Dpp target genes are well studied in wing DP. Dpp controls target genes (*sal* and *omb)* expression indirectly through repression of the transcriptional repressor Brk^24^,^26^,^27^. Dpp target gene expression patterns have not been studied in wing PE to date. The above results revealed that pMad is ubiquitously present in both DP and PE (Fig. 1B–B″). However, *brk*-*lacZ* was still present in PE (Fig. 1C–C″). Either suppressing *brk* or elevating Dpp signalling by expressing *tkv*^*QD*^ cannot induce Sal and Omb in the PE (Fig. 1H–I″ and Fig. S-1). Except for Lin (Fig. 2K)^18^, other factors, such as Bowl, Wg, and EGFR, cannot induce Sal in the PE (Fig. S-2). The factors that suppress *sal* in the PE require further investigation.

### Absence of *sal* in the PE is necessary for PE morphogenesis

As *omb* and *sal* are expressed only in the DP, not the PE, we therefore asked whether this asymmetric transcription of *omb* and *sal* is essential for correct PE formation. To test this possibility, we mis-expressed *sal* in the PE using the Gal4-UAS system. *dpp*-*Gal4* line is expressed in narrow stripes in the lateral PE (Fig. 2B, arrowhead) and in the middle DP. When *sal* was ectopically expressed in the *dpp*-*Gal4* domain, the height of lateral PE was notably elongated to a height similar to that of intermediate cells (Fig. 2A′, arrowhead) compared with control (Fig. 2B, arrowhead). Then, we expressed *sal* driven by *C765*-*Gal4* which is ubiquitously expressed in both DP and PE. A similar elongation phenotype was observed in the PE. The cell height of the central PE was elongated to a height similar to that of cuboidal cells (Fig. 2C′, arrowhead). The extent of elongation as a result of *dpp-Gal4* was stronger than that of *C765-Gal4*. This difference might be due to the differences in Gal4 activity because *dpp-Gal4* is stronger than *C765-Gal4*. The quantification of cell height (Fig. S-3) using the ratio between PE and DP cells within one wing disc revealed a significant increase in *sal* mis-expression discs (Fig. 2G). Consistently, the PE height ratio between *sal* mis-expression and control also revealed a significant increase (Fig. 2H). To confirm this result, we generated *sal* over-expression clones in PE (Fig. 2I, arrowheads). From the *x-z* cross view, the clonal height was apparently elongated to a height similar to that for cuboidal cells (Fig. 2I′, arrowhead). Therefore, we conclude that *sal* is sufficient to elongate PE height.

Unlike the effect of *sal* mis-expression, the elongation of PE height in case of *omb* ectopic expression was not apparent (Fig. 2E′,F′, arrowheads), however, the differences in the PE/DP ratio and normalized PE height for *C765*>*omb* wing discs are statistically significant (Fig. 2G,H). Strong overexpression of *omb* induces severe extrusion and basal delamination, and cell motility can thicken the wing disc^28^. Although we used a relatively weaker *UAS-omb* line, the side effect from cell movement may remain, thus leading to the statistic difference in the PE measurement. A previous report demonstrates that if Dpp signalling is suppressed in the PE by *Ubx-Gal4* driven *dad*, a portion of the PE cells are elongated to a cuboidal shape^8^. Therefore, suppressing Dpp signalling in the PE and expressing *sal* in the PE exhibit similar effects. When carefully assessing the *Ubx-Gal4* expression domain, a portion of the expressing cells were, surprisingly, located in the DP (see Fig. S-4). Thus, a non-autonomous effect from a loss of Dpp signalling in the DP in the *Ubx*>*dad* wing disc is reasonable. Because when *dad*-expressing clones were generated within PE, the height of PE did not increase (Fig. S-5). When Omb-RNAi was driven by *hh-Gal4* which is expressed in PE and the posterior compartment of DP, the posterior DP height was reduced. Interestingly, the height of opposite PE was increased (Fig. 2M,M′, arrowheads). Thus, it is possible that there is a connection between DP and PE during cell morphogenesis. To directly confirm the non-autonomous effect on PE elongation, the DP height was shortened by expression of either *dad* or *brk* within the DP specific *nub-Gal4* domain. Consistently, the PE height was apparently increased (Fig. 2N–O′, arrowheads).

### *sal* mediates the role of *lin* in the elongation of PE height

Previous studies have revealed that mis-expressing *lin* induces ectopic *sal* expression in the PE^18^. Thus, *sal* may mediate *lin*’s role in PE elongation. First, we repeated the experiment of *lin* mis-expression in the PE and consistently observed the elongation phenotype in the PE (Fig. 2K, arrowhead). Then, we revealed the transcription state of *sal* using a *sal*-*lacZ* reporter. *sal* was apparently transcribed in the PE (Fig. 2K, arrowhead). The *sal* gene complex is composed of two functionally redundant genes: *spalt major* (*salm*) and *spalt-related* (*salr*)^29^. We subsequently performed a rescue experiment by co-expressing *lin* and *salm*-*RNAi*. The morphology of the wing imaginal discs was rescued to an approximate normal state, and PE height was no longer elongated (Fig. 2L, arrowhead). We also measured the cell heights of the corresponding genotypes. *sal* down-regulation largely rescued the abnormal cell height induced by *lin* mis-expression (Fig. S-6). These results indicate that *sal* mediates the role of *lin* in promoting PE elongation. However, Lin elongated PE to a greater extent than Sal did, according to the statistic measurements. Other mediators may be involved downstream of Lin. Since that *Ubx-Gal4* line is also expressed in part of the DP cells (Fig. S-4), potential non-autonomous effects between DP and PE can not be ruled out.

### *sal* affects DP height

**Figure 3:**
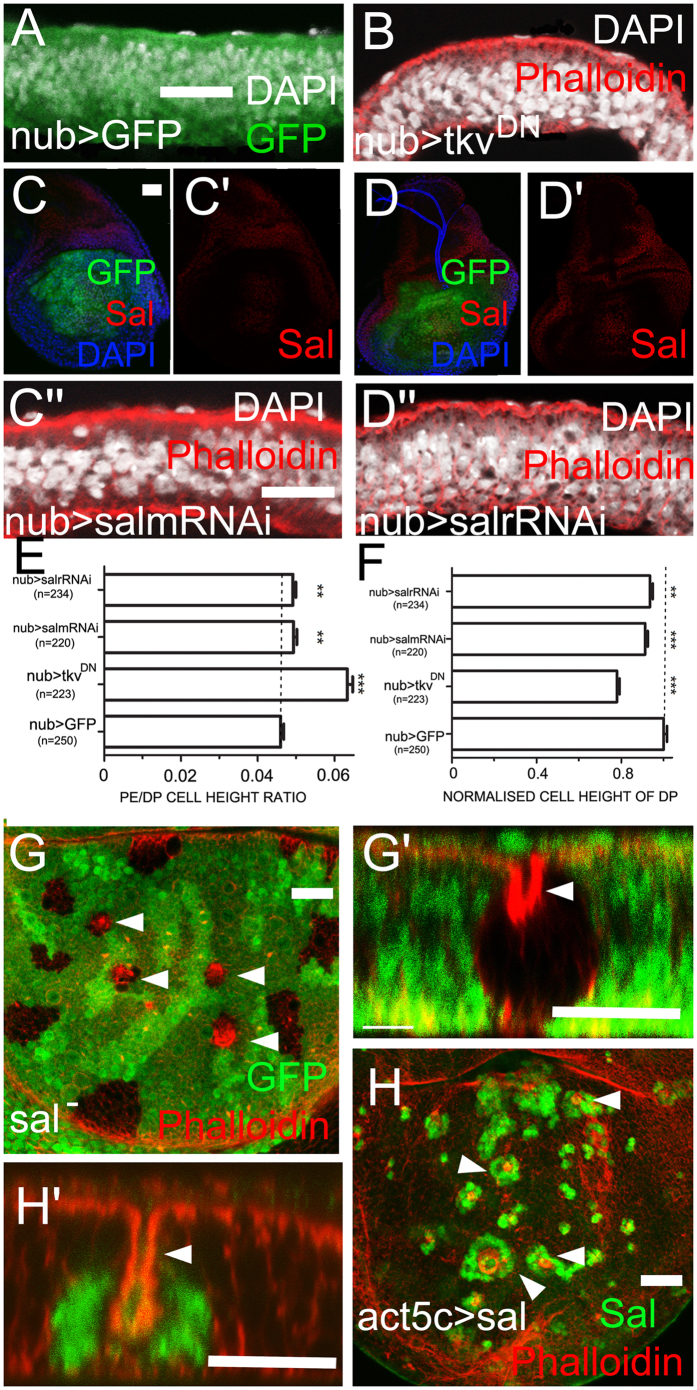
s*al* affects DP height. (**C**,C′, **D**,D′, **G**,**H**) are *x*-*y* views. Other images are *x*-*z* views. (**A**) GFP expressing control. (**B**) Expression of *UAS-tkv*^*DN*^reduces DP height. (**C**–C″) Expression of *salm-RNAi* (*nub*>*salm-RNAi*) reduces Sal levels in the DP (**C** and C′) and slightly reduces DP height (C″). (**D**) Expression of *salr-RNAi* (*nub*>*salr-RNAi*) reduced Sal levels in the DP (**D** and D′) and slightly reduces DP height (D″). The changes in PE height are not apparent but are statistically significant based on the PE/DP height ratio (**E**,**F**). (**G**) *sal* mutant clones (absence of GFP, arrowheads) in the medial DP undergo apical retraction (G′, arrowhead). (**H**) *sal*-overexpressing clones (absence of GFP, arrowheads) in the medial DP undergo apical retraction (H′, arrowheads).

Given that the mis-expression of *sal* in the PE elongates cell height, we assessed whether down-regulating Dpp-Sal signalling in DP is sufficient to shorten the DP. *nub*-*Gal4* is only expressed in the wing pouch region in the DP^7^,^18^,^30^. When Dpp signalling was mildly inhibited by expressing a dominant negative form of the Dpp receptor, *tkv*^*DN*^, in the *nub*-*Gal4* domain, DP cell height was reduced (Fig. 3B,E,F). Unlike the strong inhibition of Dpp signalling by expressing *dad* (Fig. 2N), the non-autonomous effect on PE height was not apparent in *nub*>*tkv*^*DN*^. The Dpp signalling activities in discs of *nub*>*tkv*^*DN*^*and nub*>*dad* were revealed by anti-Sal staining (Fig. S-7). The DP height was slightly reduced when *sal* was down-regulated either by *salm-RNAi* or *salr-RNAi*; however, the extent of this reduction was weaker than that of *tkv*^*DN*^ (Fig. 3C–F). Then, we generated *sal* mutant clones marked by the loss of GFP in the DP. The intensity of F-actin labelled by Phalloidin was much stronger in the clone regions (Fig. 3G, arrowheads). The *x-z* cross-section showed that the apical side of *sal* mutant clones in the DP was retracted toward the basal side (Fig. 3G′, arrowhead). These data suggested that Dpp-Sal signalling is required to maintain DP elongation. Interestingly, a similar retraction phenotype was also observed in *sal*-overexpressing clones (Fig. 3H,H′). Therefore, both *sal* loss- and gain-of-function clones induce an apical retraction phenotype in the DP^31^. This phenotype is observed in both *omb* loss- and gain-of-function clones in the DP^14^. Omb exhibits a graded distribution in the DP along the A/P axis and specifies unknown apically distributed adhesion molecules. A continuous Omb level is essential for maintaining the epithelial integrity of the wing disc^14^. Therefore, sharp discontinuity in either Omb or Sal levels in the DP induces apical retraction of cells. To confirm this conclusion, we generated a sharp discontinuity of Sal in the DP using *dpp-Gal4* driven *UAS*-*sal*. Sal continuity was disrupted at the A/P boundary and where deep apical folds were formed (Fig. S-8). The expression domain of *sal* in the DP is narrower than that of *omb* and *vg*. Beyond the *sal* domain, *omb* and *vg* can ensure the correct cell morphogenesis in the DP. Clones lacking Vg function are also extruded from the DP layer^32^.

### Mis-expressing *sal* converts the PE into a cuboidal shape

**Figure 4:**
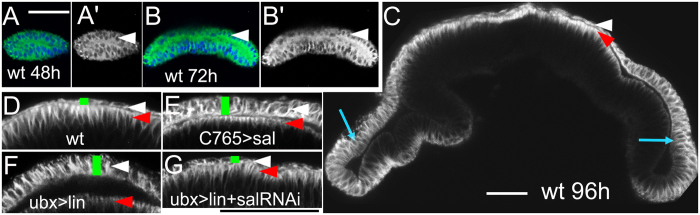
Microtubule dynamics during cell height change. All the images are *x*-*z* views. Stains are anti-α-tubulin and DAPI. White arrowheads indicate the PE layers. Red arrowheads indicate the apical side of DP cells. Blue arrows indicate the intermediate cells. Green bars in (**D–G**) indicate the cell height of PE. (**A–C**) Time course of microtubule dynamics in wild type control wing discs. PE height starts to flatten at the early L3 stage (**B**, arrowhead). Microtubules are polarized to the apical side of the DP (**C,D**, red arrowheads) compared with the even distribution in PE (**C,D**, white arrowheads) and intermediate cells (**C**, blue arrows). (**E**) Microtubules are enhanced in the PE (white arrow) when *sal* is expressed. (**F**) *lin* expression induces similar microtubule changes upon *sal* expression. Note that the cell height and microtubule levels of the PE (**E**,**F**, white arrows) are similar to that of intermediate cells (**C**, blue arrows). (**G**) Co-expression of *sal-RNAi* and *lin* rescues PE height and microtubule levels similar to that noted in wild type.

Microtubule cytoskeleton is polarized during cell morphogenesis in the wing imaginal disc^33^. To reveal microtubule-based cytoskeleton changes induced by either *lin* or *sal* mis-expression and the microtubule dynamics during normal development, we monitored the microtubule level via antibody staining. In the 2^nd^ instar, all cells were cuboidal shape, and microtubules were uniformly distributed (Fig. 4A, arrowhead). During the early 3^rd^ instar, the cell shape begins to differentiate. PE cells were largely shortened (Fig. 4B,C, white arrowheads), whereas DP cells were remarkably elongated^8^,^9^ (Fig. C, red arrowhead). Correlating with DP elongation, the microtubule network was asymmetrically enriched to the apical side of the DP (Fig. 4C,D, red arrowheads). When *lin* was mis-expressed in the PE by *Ubx-Gal4*, *sal* was activated in the PE (Fig. 2K). Both direct and indirect *sal* expression (Fig. 4E,F) induced PE height elongation (comparison between green bars indicated PE height in Fig. 4D–F) with an even microtubule distribution (Fig. 4E,F white arrowheads). The microtubule levels of PE were increased compared with the wild type PE (comparison between white arrowheads indicated positions in Fig. 4D–F). The microtubule distribution in Sal-elongated PE was similar to that in very lateral PE and intermediate cells (Fig. 4C, blue arrows) in the L3 stage or undifferentiated cells in earlier larval stages (Fig. 4A, arrowhead). In the rescue experiment, *lin* and *salm-RNAi* were co-expressed. PE height was restored (Fig. 4G, green bar), and the microtubule levels in the PE (Fig. 4G, white arrowhead) were similar to that in wild type PE (Fig. 4D, white arrowhead). Therefore, based on the cell height and microtubule distribution, Sal mis-expression converts the PE into a cuboidal cell shape.

During tissue morphogenesis, cell-shape changes always accompany microtubule cytoskeleton rearrangement^33^,^34^,^35^,^36^,^37^. Dpp signalling activity has been proposed to play a basic function in microtubule organization. Dpp signalling is graded in the DP along the A/P axis, with higher levels in the medial DP region, which is enriched in apical microtubules^12^,^13^. Thus, a correlation is noted between Dpp signalling activity and microtubule levels in the DP. Clones with loss-of-function of Dpp receptors in the DP appear extruded (cell height is severely shortened) and exhibit reduced apical microtubule levels^12^,^13^. Consistently, clones with both loss- and gain-of-function *sal* (Fig. 3 and Fig. S-9) and *omb* in the DP consistently exhibit severe apical retraction with shortened cell height and loss of apical microtubule enrichment^14^. Therefore, our data support the hypothesis that Dpp-Omb/Sal signalling activity plays a more general function in microtubule-based cell morphogenesis. Other transcription factors that induce reductions in DP cell height also correlate with the loss of apical microtubule enrichment. The Tbx6 subfamily gene cluster *Dorsocross* (*Doc*) initiates wing hinge/blade fold formation. In the Doc expression domain in the DP, cells are shortened from the apical side with severe loss of apical microtubules^38^.

## Methods

### *Drosophila* stocks

The mutant allele was deficiency *Df(2 L)32FP-5*, which removes both *sal* and *salr*^39^. The following transgenes were used: *dpp-Gal4*^40^, *nub-Gal4*^30^, *C765*-*Gal4*^41^, *Ubx-Gal4*^42^, *UAS-GFP* (Bloomington Stock Center), *UAS-CD8-GFP*^43^, *UAS-salm*^22^, *UAS-omb*^23^ (weaker line omb595), *UAS-lines*^44^, *UAS-tkv*^*DN*^^45^, *UAS-tkv*^*QD*^^41^, *UAS-dad*^46^, *UAS-brk*^27^, *UAS-wg* (Bloomington Stock Center), *UAS-EGFR*^*CA*^^47^, *UAS-salm-RNAi* (VDRC 3029), *UAS-salr-RNAi* (VDRC 28386), *UAS-brk-RNAi* (VDRC 2919), *UAS-bowl-RNAi* (VDRC 18428), and flip-in *AYGal4*^48^. Enhancer trap lines used were *sal-lacZ*^49^, *omb-lacZ*^23^ and *brk*^*X*47^*-lacZ*^26^.

### Transgene expression and clone generation

Larvae were raised at 25 °C. For the efficient expression of RNAi and UAS transgenes driven by the weak *C765*-*Gal4*, larvae were raised at 29 °C.

Marked clones of mutant cells were generated by Flp-mediated mitotic recombination^50^ and by subjecting 1^st^ instar larvae to a 35.5 °C heat-shock for 30 min. Transgenes were expressed using the Gal4–UAS system^51^.

Larval genotypes for clone generation:

y w hs-flp; ubi-GFP FRT40/*Df(2 L)32FP-5* FRT40

y w hs-flp; act5c>CD2>GAL4/UAS-GFP; UAS-tkv^QD^/+

y w hs-flp; act5c>CD2>GAL4/UAS-CD8-GFP; UAS-salm/+

y w hs-flp; act5c>CD2>GAL4/UAS-CD8-GFP; UAS-dad/+

### Immunohistochemistry

Dissected wing imaginal discs were fixed and stained with antibodies according to standard procedures. The following primary antibodies were used: rabbit anti-pMad, 1:200 (Cell Signalling); mouse anti-α-Tubulin, 1:2000 (Sigma); rabbit anti-β-galactosidase, 1:2000 (Promega); mouse anti-Ubx, 1:200 (DSHB); rabbit anti-Omb, 1:1000 and rabbit anti-Sal, 1:500 (a gift from Coralia Pérez Fernández); mouse anti-EGFR, 1:200 (abcam); and mouse anti-Wg 1:200 (DSHB). Secondary antibodies (diluted 1:200) included goat anti-mouse DyLight 488 and goat anti-mouse DyLight 549 (Agrisera) and goat anti-rabbit DyLight Cy5 (Jackson ImmunoResearch). Cell nuclei were stained with DAPI (1:500, Sigma). F-actin was visualized with Rhodamine-phalloidin, 1:2000 (Sigma). Images were collected using a Leica TCS SP2 AOBS confocal microscope. Cell height was calculated based on high resolution confocal images using the Image-J program.

### Wing disc cryosectioning

After secondary antibody staining, discs were re-fixed for 30 minutes in 4% paraformaldehyde, washed, and stored in 30% sucrose solution at 4 °C overnight. Discs were oriented in Tissue-Tek (Sakura Finetek), frozen and cut into 20-μm sections on a cryostat (YD-1900, YIDI, China).

## Additional Information

**How to cite this article**: Tang, W. *et al*. Asymmetric distribution of Spalt in *Drosophila* wing squamous and columnar epithelia ensures correct cell morphogenesis. *Sci. Rep.* **6**, 30236; doi: 10.1038/srep30236 (2016).

## Supplementary Material

Supplementary Information
