# Supplementary material for: Asymmetric distribution of Spalt in Drosophila wing squamous and columnar epithelia ensures correct cell morphogenesis
Source: Sci Rep. 2016 Jul 25;6:30236. doi: 10.1038/srep30236 (PMC4958983; doi:10.1038/srep30236)
Supplement: Supplementary Information [file srep30236-s1.pdf]

Asymmetric distribution of Spalt in *Drosophila* wing squamous and columnar epithelia ensures correct cell morphogenesis

Wenqian Tang, Dan Wang, Jie Shen*

Department of Entomology, China Agricultural University, 100193 Beijing, China

* Correspondence: shenjie@cau.edu.cn

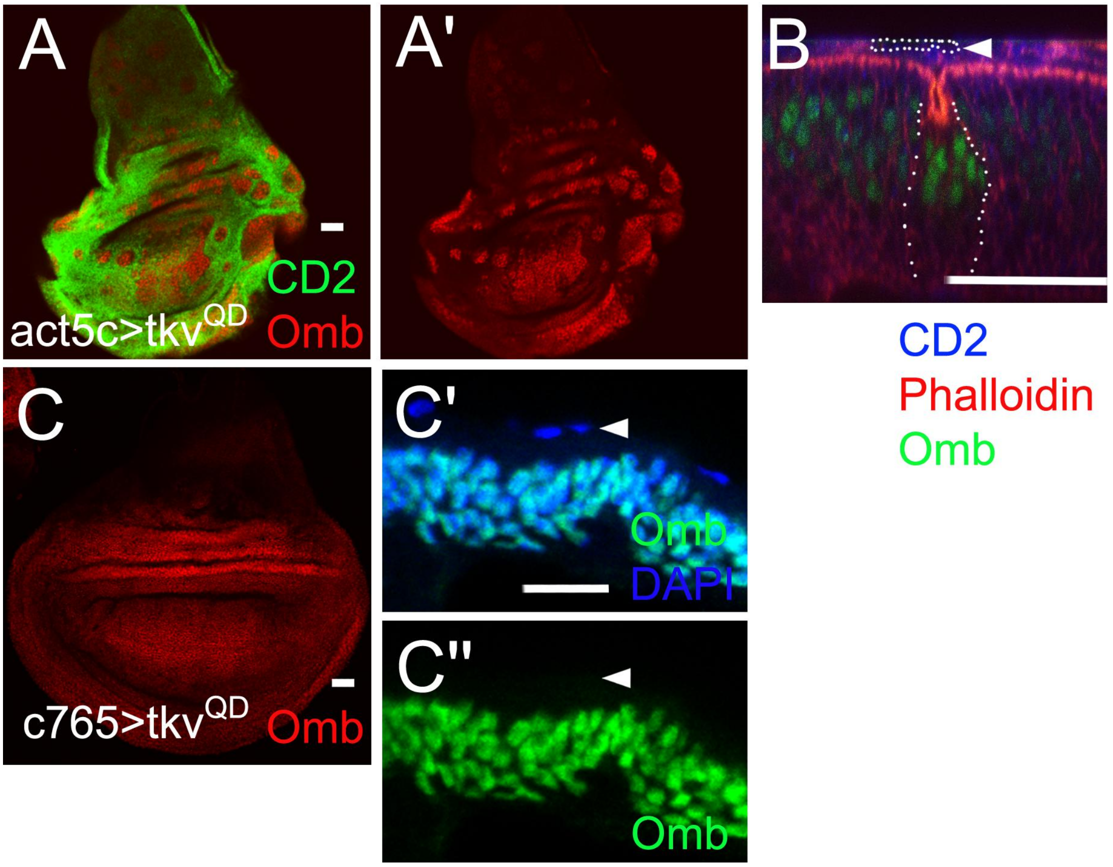

Fig. S-1 *omb* is not induced by Dpp signalling in the PE

(A) The expression of tkv^{QD} in clones induces Omb in the DP (A') but not in the PE (B, arrowhead in *x-z* view). (C) Ubiquitous expression of tkv^{QD} induces Omb in the DP but not in the PE (C' and C'', arrowheads in *x-z* view).

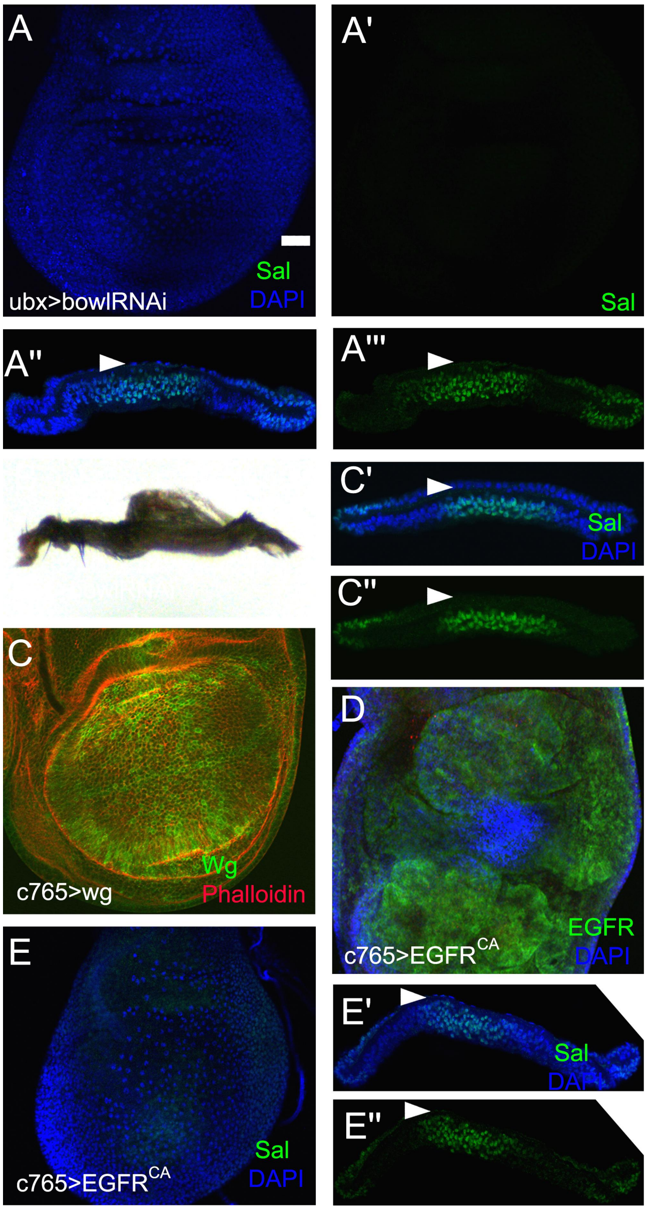

Fig. S-2 No genetic interactions among *sal* and *bowl*, *wg*, and EGFR.

(A-A'') Suppression of *bowl* does not induce Sal in the PE (arrowheads). A and A' are focused on the PE plane. (B) Suppression of *bowl* induces severe defects in adult wing. (C-C'') Expression of *wg* does not induce Sal in the PE (arrowheads). (D) Expression of $EGFR^{CA}$ induces overgrowth and severe distortion of the disc at the late L3 stage. (E) To avoid the severe distortion in D, middle L3 wing discs are selected. Sal is not induced in the PE (E' and E'', arrowheads) by $EGFR^{CA}$ expression.

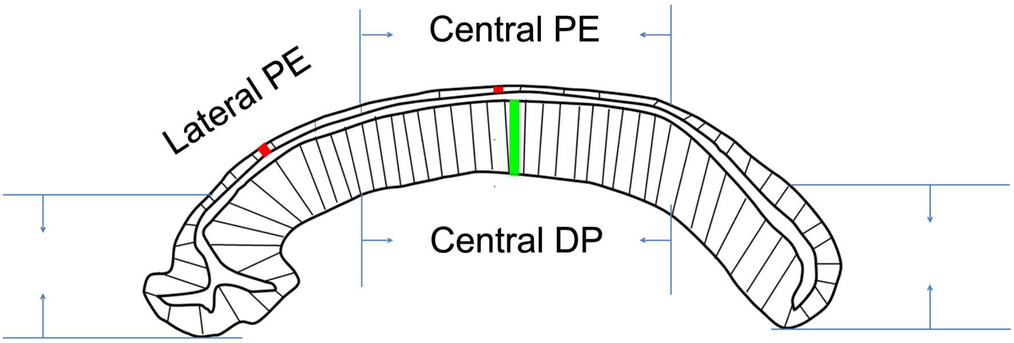

Fig. S-3 A schematic drawing to illustrate how to measure the height of the PE and DP.

Based on high-resolution Phalloidin images, draw a line at the red bar from the apical side to basal side of PE or lateral PE (for *dpp-Gal4* experiments) and a line at the green bar for DP. The length of the line was measured using the Image-J program and reported with arbitrary units.

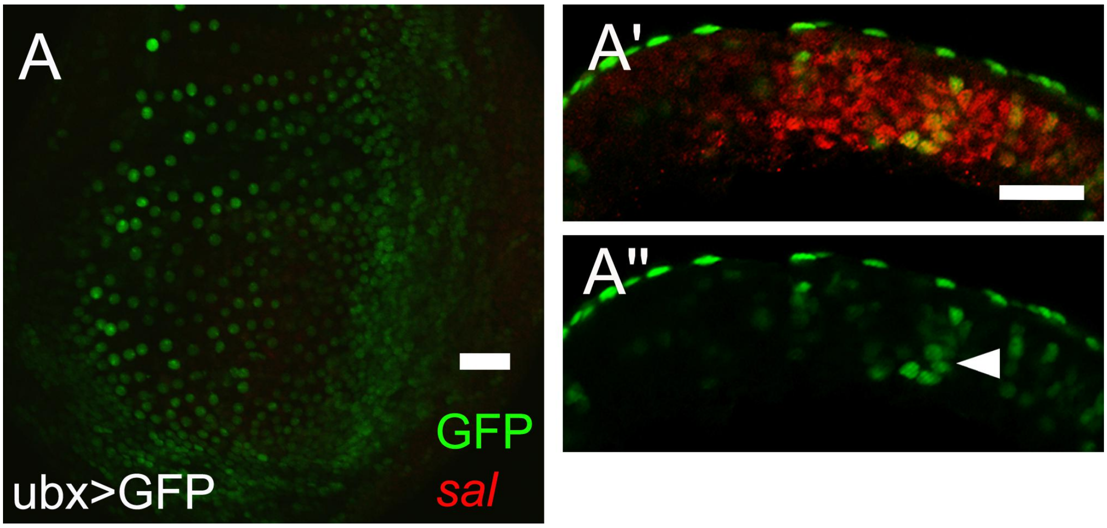

Fig. S-4 When UAS-GFP is expressed in the *ubx-Gal4* domain, GFP is mainly expressed in PE cells (A) and in some DP cells (A' and A'', arrowhead).

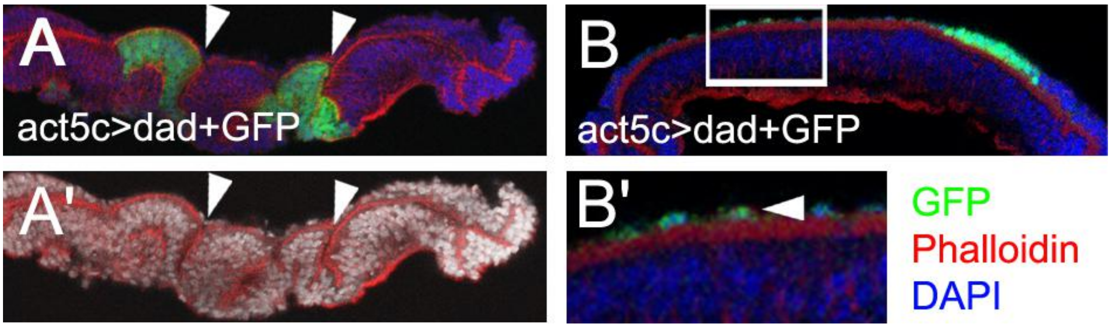

Fig. S-5 Clones expressing *dad* induce fold or invagination in DP (A and A’, arrowheads) but not affect PE cell morphology (B and B’, arrowhead).

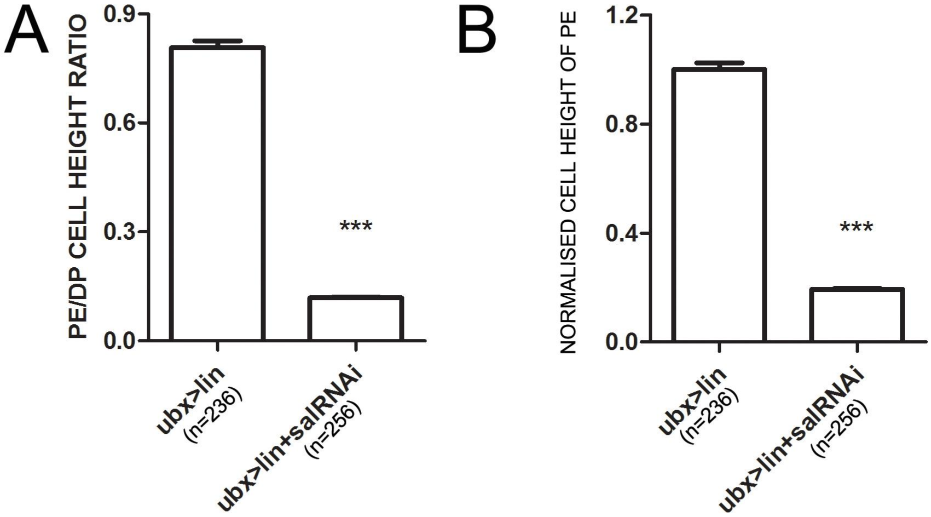

Fig. S-6 Statistical comparison of PE height.

Expression of *sal-RNAi* largely rescues *lin*-induced PE elongation, which was statistically compared with the PE/DP height ratio (A) and the normalized PE height (B).

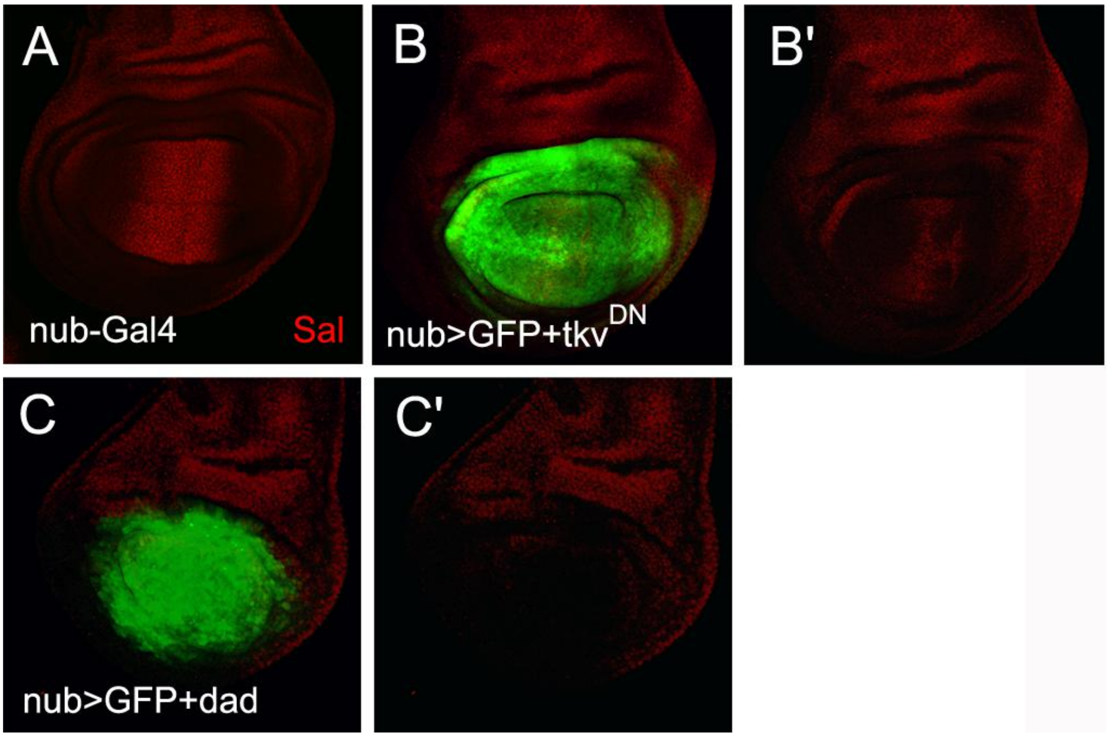

Fig. S-7 Expressing a dominant negative form of the Dpp receptor, *tkv*^{DN}, in the *nub-Gal4* domain partially suppresses Dpp target gene *sal* expression (B and B’). While expressing *dad* strongly suppresses Sal within the *nub-Gal4* domain (C and C’).

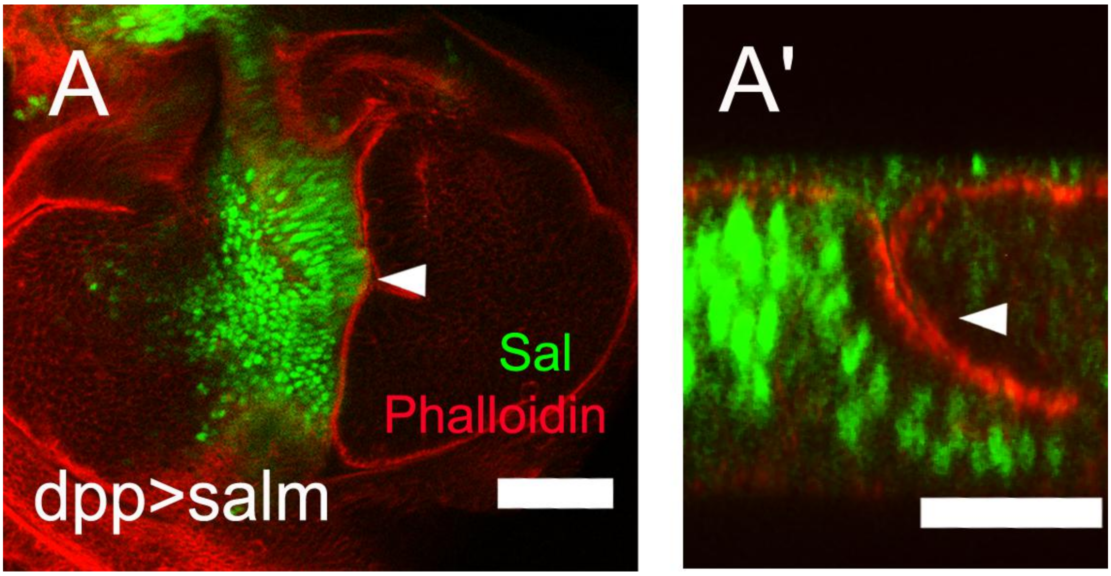

Fig. S-8 Expression of *sal* in the *dpp-Gal4* domain induced an additional fold along the A/P boundary (A, arrowhead). When assessed using a *x-z* view, the fold is composed of apical retracted cells (A', arrowhead).

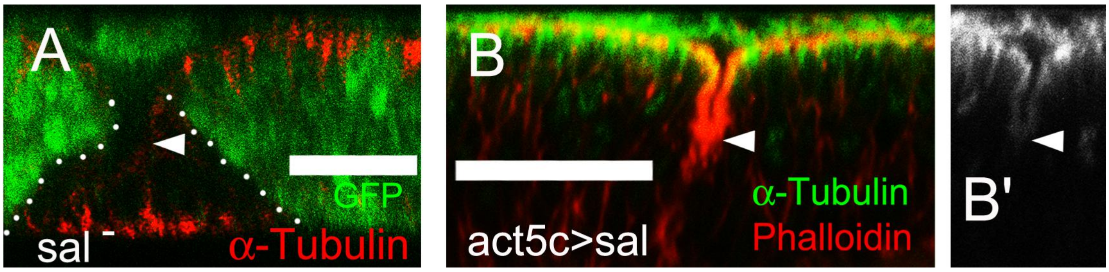

Fig. S-9 Apical microtubule enrichment in the DP is lost in *sal* loss- and gain-of-function clones.

The outlines of PE and DP are visualized by Phalloidin-labelled F-actin. (A) *sal* mutant clones exhibit reduced apical microtubule enrichment (arrowhead). (B) *sal*-overexpressing clones exhibit reduced apical microtubule enrichment (arrowhead). *sal*-overexpressing clones in the DP are consistently retracted from the apical side (see Fig. 3 H and H'). Thus, we assume that the Phalloidin-marked apical retraction indicates the apical side of the clones.
